# Comparison between bearing strengths of molded-in and machined holes of GFR/PP composites

**DOI:** 10.1038/s41598-022-18943-w

**Published:** 2022-08-30

**Authors:** M. M. Osama, A. I. Selmy, Ayman M. M. Abdelhaleem, A. A. Megahed

**Affiliations:** 1 Mechanical Engineering Department, Higher Technological Institute, Tenth of Ramadan city, Al-Sharqia Egypt; 2 Mechanical Design and Production Engineering Department, Faculty of Engineering, Zagazig University, P.O. Box: 44519, Zagazig, Al-Sharqia Egypt

**Keywords:** Materials science, Composites, Mechanical properties

## Abstract

This study is an investigation of weight fraction (wt%) and fiber feedstock length (FFSL) effects on the bearing strength (BS) of bolted joints in glass-fiber-reinforced (GFR) polypropylene (PP) composites manufactured by an injection molding technique. The investigation was made for holes produced either by molding or machining. For machined holes, the effect of drilling parameters (feed and speed) on BS was discussed. It is observed that BS decreased as FFSL increased. BS of both molded-in and drilled specimens was enhanced by increasing wt% of glass fiber. While slightly better BS was observed for molded-in specimens than drilled ones for all specimens. The drilling conditions’ effect on BS was found to be insignificant for drilled holes in long fibers reinforced PP, where the most significant factor was wt%. However, for short fibers reinforced PP, the spindle speed was the most significant factor followed by feed, while wt% has the lowest effect. Failure morphology mode for specimens indicates that for molded-in specimens, neat PP specimens failed under pure bearing mode while GFR/PP specimens failed under the mixed-mode failure (bearing and net tension). For machined specimens, all specimens failed under mixed-mode failure except for the highest wt% specimens which failed under net tension.

## Introduction

Recently the usage of thermoplastic materials has been increasing steadily as they provide a unique collection of properties. Strength-to-weight ratios, environmental resistance, rapid processing, superior high-temperature performances, and recyclability are some of thermoplastics’ advantages that favor their use over other materials^1–3^. The addition of fibers is widely used in reinforcing polymeric-based composites to be more reliable in their applications. In order to use fiber-reinforced polymers (FRPs) as a structural element, these materials have to fulfill some requirements such as stiffness, strength, durability, impact and crush to be more useful in the manufacturing and assembly of critical components^2^. Various automobile parts have been produced using fiber-reinforced thermoplastics, as their lightweight effects have been demonstrated by non-load bearing^4^ and semi-load bearing parts. These parts include battery boxes^5–7^, crash boxes^8^, lightweight wheels^9^, front-end modules^10^, automotive seats^11,12^, leaf springs^13^, and hoods^14^. In particular, GFR/PP appears to have a good potential for application in the building of prefabricated structures such as houses, barriers, beams, and bridge decks in the field of civil engineering^15^. Vaidya and Chawla^16^ designed and fabricated a durable bus seat made of GFR/PP providing 43% and 18% savings, respectively, in weight and total production cost compared to commonly used seat designs.

The joints represent one of these critical components where bolts provide the primary means of connecting FRPs in the structural application, construction of aircraft, aerospace, automotive vehicles, and other engineering applications with high performances including sporting goods, wind energy structures, and medical appliances^17–23^. The joint strength of GFR/PP was found to be suitable for the design of the leaf spring and hence these types of materials can be utilized for joint applications^24–26^. Also, Anandakumar et al.^27^ obtained a superior performance from GFR/PP control arm, as a load-bearing component of the suspension system, in comparison to steel. The joint design has a particular interest in FRPs structures since joints represent the weakest point in a composite structure and the composite material capability of redistributing local high stresses through yielding^20^. BS is an important property that must be taken into consideration in joint design.

The holes needed for joining FRPs are usually manufactured by the drilling process which weakens the reinforcement structure. Nejhad and Chou^28^ explained that drilled holes significantly reduced the performance of Carbon fiber/epoxy composite due to a through-the-thickness cut of fibers which can be averted by using molded-in holes. Nejhad and Chou^29^ considered that for all practical purposes, a molded-in hole is more desirable than a drilled hole. Therefore, many alternative hole molding techniques were developed. Hufenbach et al.^30^ used a technique based on fiber shifting which enables non-destructive manufacturing of the holes. Brookstein and Tsiang^31^ found that integrally-formed braided holes provide a 180% increase in joint BS compared with machined holes in the graphite fibers/epoxy composite. Chang et al.^32^ showed in a study of mechanical joining by pin load of Kevlar/epoxy, Graphite/epoxy, and Kevlar-Graphite/epoxy hybrid composites that specimens with molded-in holes gave 0.12–61.23% strength enhancement compared with drilled hole specimens.

Experimental results of Lin et al.^33^ showed that there exists larger failure strength, smaller initial stiffness, and larger failure strain of woven glass roving composite (0,90)_s_ with a molded-in hole relative to those with a drilled hole. However, for (45, − 45)_s_ there is no improvement for failure strength and initial stiffness except for failure strain. Zitoune et al.^34^ observed that molded-in holes of woven carbon fibers/epoxy composite exhibit 30% higher strength and 100% lower strain than drilled holes. Brown et al.^35^ showed that for advanced manufacturing of carbon fiber/PEEK thermoplastic composites, better open-hole tensile and compressive properties were obtained when holes were produced by thermally assisted piercing technique as compared with drilled holes. Fujita et al.^36^ found that for the braided glass fiber reinforced epoxy composite (GFRE), the joint strength of the braided hole was greater than that of the machined hole. Also, Herszberg et al.^37^ found that weft-knitted and woven glass fiber/epoxy composites with integrally formed holes had a BS about 20% greater than those with drilled holes. Durante and Langella^38^ found a high BS of the GFRE composite specimens with the molded-in hole, made by shifting the fibers around the hole, compared to the BS of the specimens with holes made by cutting the fibers by drilling. Dickson and Dowling^39^ found that the BS of 3D printed Carbon fiber/Nylon composite with a drilled hole is lower than that of the ‘Tailor Woven’ integrated hole by 63% if it goes under double shear testing. Clark^40^ concluded that the average increases in bearing stress and bearing strain at failure of cut carbon fibers/Nylon composites for the printed-in holes (allowing for fiber orientation circumferentially around the hole) compared with the drilled-in holes were 31% and 86.8%, respectively.

On contrary, Ataş et al.^41^ concluded that the BS of the Tri-axial braided carbon fiber/epoxy specimens with a molded-in hole was reduced than specimens with drilled holes, due to the increased misalignments of the fibers during the manufacturing process. Wang’s^42^ comparison between holes produced by tri-axial braided glass roving/epoxy specimens and machined holes indicated that the braided hole showed similar or even lower bearing capacity compared with the machined hole.

For machined holes, the BS of FRPs with drilled holes was mainly affected by the machining conditions. Khashaba et al.^19–21^ found that specimen stiffness and BS of GFRE decreased as the feed rate and cutting speed increased. Khashaba and El-Keran^43^ observed a lower BS of woven GFRE composites that were drilled at a speed of 16.3 m/min compared with drilling at 32.7 m/min while feed values had an insignificant effect on BS at speed of 16.3 m/min but it had a clear effect at 32.7 m/min (increased then decreased). Krishnaraj et al.^44^ illustrated that drilling at a rotational speed of 3000 rpm and a feed rate of 0.02 mm/rev led to the highest BS compared to other spindle speeds and feeds. Tagliaferri et al.^45^ concluded that for a given drilling speed to feed rate ratio, better results in terms of BS may be obtained by adopting a lower drilling speed for GFRE specimens. Srinivasa Rao et al.^46^ found that small feed rates are preferred in the drilling of woven GFRE composite laminates. Wang et al.^47^ indicated that both rotational speed and feed when drilling GFRE laminates had an insignificant effect on the BS.

Studies dealing with the drilling of thermoplastic matrix composites cover some aspects where Ilio et al.^48^ discussed the damage caused by drilling in unidirectional composites made with thermoplastic matrix reinforced with graphite fibers concerning different machining parameters. Hocheng and Puw^49,50^ demonstrated that the carbon-fiber-reinforced Acrylonitrile Butadiene Styrene (ABS) composites had good machinability in drilling compared with epoxy-based composites. Mudhukrishnan et al.^51^ discussed the effect of drill material, spindle speed, and feed rate on delamination and thrust force on woven glass fabric reinforced polypropylene laminates.

Manufacturing parts with readymade holes requiring no further processing distinguishes injection molding from other manufacturing techniques. Injection molding can flexibly provide thermoplastic constructions reinforced with short fibers as an enhancement for strength including joint strength. However, there are limits to joint strength enhancement through increasing shot fiber content^52^. Despite reinforcing injection molded parts with continuous fibers is not possible, holes could be reinforced using embedded local continuous fibers^52^. Moreover, an allowance of improving joint performance by metallic insert is possible through injection molding^53,54^, where direct adhesion between the plastic and the metal could be secured^55^.

From the previous literatures it has been clear, according to our knowledge, that limited research works are concerned with studying the effect of weight fraction on the BS of injection-molded chopped glass fiber reinforced thermoplastic composites**.** Also, rare studies were carried out in the comparison between the BSs of molded-in holes and drilled holes of this type of material. Moreover, there is no systematic study carried out to indicate the effect of machining conditions on the BS for such type of materials. Accordingly, the present work aims to study the effect of change in weight fraction of glass fiber on the BS of injection-molded chopped glass fiber reinforced thermoplastic (PP) composites. An investigation was made to compare between the BS of molded-in and drilled holes of these type of composites. Moreover, the influence of drilling parameters (feed rate, spindle speeds) on BS of specimens with machined holes was studied.

## Materials and experimental work

### Materials

The matrix material used in this work was Polypropylene (PP) copolymer for injection molding (413MNK45) which was supplied by SABIC^®^—Egypt. Glass fiber (GF) used in the present work was E-glass chopped strands with filament chop lengths of 12 and 24 mm and were supplied by JUSHI Co. Mechanical and physical properties of GF and PP are presented in Tables 1 and 2, respectively.

**Table 1:** Mechanical and physical properties of reinforcement glass fibers (GF).

**Physical properties**	
Density	2.55 g/cm^3^
Softening point	900 °C
Filament diameter	13 µm
**Mechanical properties**	
Tensile strength @ break	1950 MPa
Elongation @ break	4.8%
Young’s modulus	72 GPa
Shear modulus	30 GPa
Ductility	0.026

**Table 2:** Mechanical and physical properties of the matrix-polypropylene (PP).

**Physical properties**	
Melt flow rate at 230 °C and 2.16 kg	70 g/10 min
Density at 23 °C	0.905 g/cm^3^
Vicat softening temperature	150 °C
**Mechanical properties**	
Tensile strength @ yield	28 MPa
Elongation @ yield	5%
Flexural modulus (1% Secant)	1550 MPa
Rockwell hardness, R-scale	94

### Specimens manufacturing

Specimens for the bearing test were manufactured using plastic injection molding using HAITIAN PL1200 injection molding machine with a maximum clamping force of 1200 KN. The mold was designed, manufactured, and examined several times to check its suitability for producing the desired specimens according to ASTM D5961 standard for bearing specimens. In this mold, the same direction of the flow for the plastic is considered for each specimen to avoid the probability of weld lines formation which may lead to cracks.

**Figure 1:**
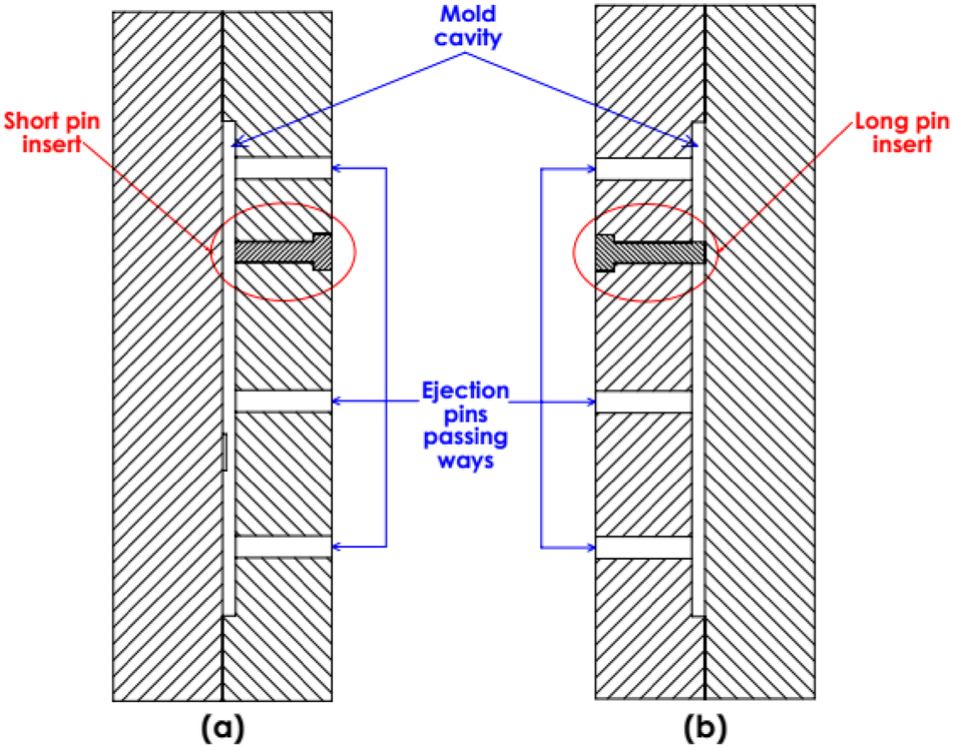
The mold; (**a**) mold with short insert, (**b**) mold with long insert for specimens with molded-in holes.

The mold is provided with two 6 mm diameter inserts; short and long inserts. The short insert is used for specimens without molded-in holes (the holes will be drilled later using the machining process), Fig. 1a, while the long insert is used for specimens with molded-in holes (the holes are produced as a result of the injection molding process) as illustrated in Fig. 1b.

To produce the test specimens, the barrel temperature profile along the injection-molding machine was adjusted to be 140, 160, 180, 220, and 244 °C. The barrel temperature was adjusted during the process when adding PP with GF. The manufacturing process can be described as follow; First, neat PP bearing specimens were injection molded. Second, PP pellets were mechanically blended with GF using different weight fractions of 10, 20, and 30 wt% of PP and different feedstocks having fiber lengths of 12 mm and 24 mm. The mixture was first fed to an extruder of the injection molding machine to produce pre-samples. Pre-samples with their sprue and runners are crushed in a crusher forming small particles of identical sizes. The small particles were injection molded once again to obtain the final test specimens. The main purpose of these stages is to gain a better distribution of the GF into the PP. The whole process is repeated with a long insert added to the mold to produce specimens with molded-in holes. The manufactured specimens had chopped and randomly oriented fibers. The codes and compositions of specimens manufactured with molded-in holes are illustrated in Table 3.

**Table 3:** Codes for specimens with molded-in holes.

Code	PP%	GF%	Feedstock fiber length (mm)
L00	100	0	–
L1012	90	10	12
L2012	80	20	12
L3012	70	30	12
L1024	90	10	24
L2024	80	20	24
L3024	70	30	24

### Drilling process

The drilling process was performed using a Boxford 300VMC_i_ CNC milling machine equipped with Boxford pc software. A manual vise is used to hold and consistently locate the workpiece. A 6 mm diameter carbide twist drill bit (as recommended in the drilling of GFR/PP by Mudhukrishnan^51^) provided by AYKT is used in the drilling process. The drilling process was conducted in dry conditions without the use of coolant. Drilling was performed with the support of a wooden plate at the back of the composite samples. The experimental factors at different levels were designed using the Taguchi method as shown in Table 4.

**Table 4:** Factors of the drilling process and their levels.

Factors	Levels			
	1	2	3	4
Weight fraction (wt%)	0	10	20	30
Spindle speed (r.p.m)	1000	2000	3000	4000
Feed rate (mm/min.)	100	200	300	400

### Design of experiments

Full factorials design of experiments identifies all possible combinations for a given set of factors. Since most industrial experiments usually involve a significant number of factors, a full factorial design results in more experiments. To minimize the number of experiments to a reasonable level, only a small group from all the possibilities is chosen. Taguchi analysis offers a unique set of design guidelines that cover several aspects of factorial experiments. The experimental design by the Taguchi method involves orthogonal arrays organizing the process parameters and possible variation levels. It determines the factors that affect product quality the most with minimized experimentations, thus saving resources and time. In the present work, the factors of the drilling process are fiber weight fraction, spindle speed, and feed rate. The levels of fiber weight fractions were 0, 10, 20, and 30 wt%. The selected levels of cutting speeds were 1000, 2000, 3000, and 4000 rpm. While levels of feed rate were taken as 100, 200, 300, and 400 mm/min. These factors and their levels are shown in Table 4.

The drilling experiments were performed using the L16 mixed orthogonal array including 16 runs corresponding to several tests for Taguchi’s method. The experimental layout is given in Table 5.

**Table 5:** Taguchi L16 orthogonal array for drilled PP and GFR/PP under different drilling conditions.

Experiment no.	Weight fraction (%)	Spindle speed (r.p.m)	Feed rate (mm/min.)	Specimen code
1	0	1000	100	W1S1F1
2	0	2000	200	W1S2F2
3	0	3000	300	W1S3F3
4	0	4000	400	W1S4F4
5	10	1000	200	W2S1F2
6	10	2000	100	W2S2F1
7	10	3000	400	W2S3F4
8	10	4000	300	W2S4F3
9	20	1000	300	W3S1F3
10	20	2000	400	W3S2F4
11	20	3000	100	W3S3F1
12	20	4000	200	W3S4F2
13	30	1000	400	W4S1F4
14	30	2000	300	W4S2F3
15	30	3000	200	W4S3F2
16	30	4000	100	W4S4F1

In the analysis of Taguchi, the values of each experiment are then converted into a signal-to-noise (S/N) ratio, where the term which refers to the required values (mean) is the signal and the values which are not required (Standard deviation) are represented as the noise for the output characteristics. When analyzing S/N ratios, the quality characteristics are proposed by Taguchi as follows^56^;

1 $$\text{Larger is better }\quad S/N \,ratio \,\left(\eta \right)=-10{\text{log}}_{10}\frac{1}{n}\sum_{i=1}^{n}\frac{1}{{y}_{i}^{2}},$$

2 $$\text{Smaller is better } \quad S/N\, ratio\, \left(\eta \right)=-10{\text{log}}_{10}\frac{1}{n}\sum_{i=1}^{n}{y}_{i}^{2},$$

3 $$\text{Nominal is best } \quad S/N \,ratio\, \left(\eta \right)=-10{\text{log}}_{10}\frac{{\mu }^{2}}{{\sigma }^{2}},$$

where ${y}_{i}$ is the observed response value and $n$ defines the number of replications.

When the goal of the experimentation is to maximize the response, selecting “larger is better” quality characteristic is the right choice (Eq. (1)). However, if the goal of experimentation is to minimize the response, selecting ‘‘smaller is better’’ quality characteristic is the right option (Eq. (2)). “Nominal is best” (Eq. (3)) is used for targeting the response to base the signal-to-noise ratio on means $(\mu )$ and standard deviations $(\sigma )$. The “Nominal is best” signal-to-noise ratio is useful for analyzing or identifying scaling factors, which are factors in which the mean and standard deviation vary proportionally. Scaling factors can be used to adjust the mean on target without affecting signal-to-noise ratios.

In the present work, the objective is to maximize the BS, therefore, “larger is better” quality characteristic is selected.

### Bearing test

**Figure 2:**
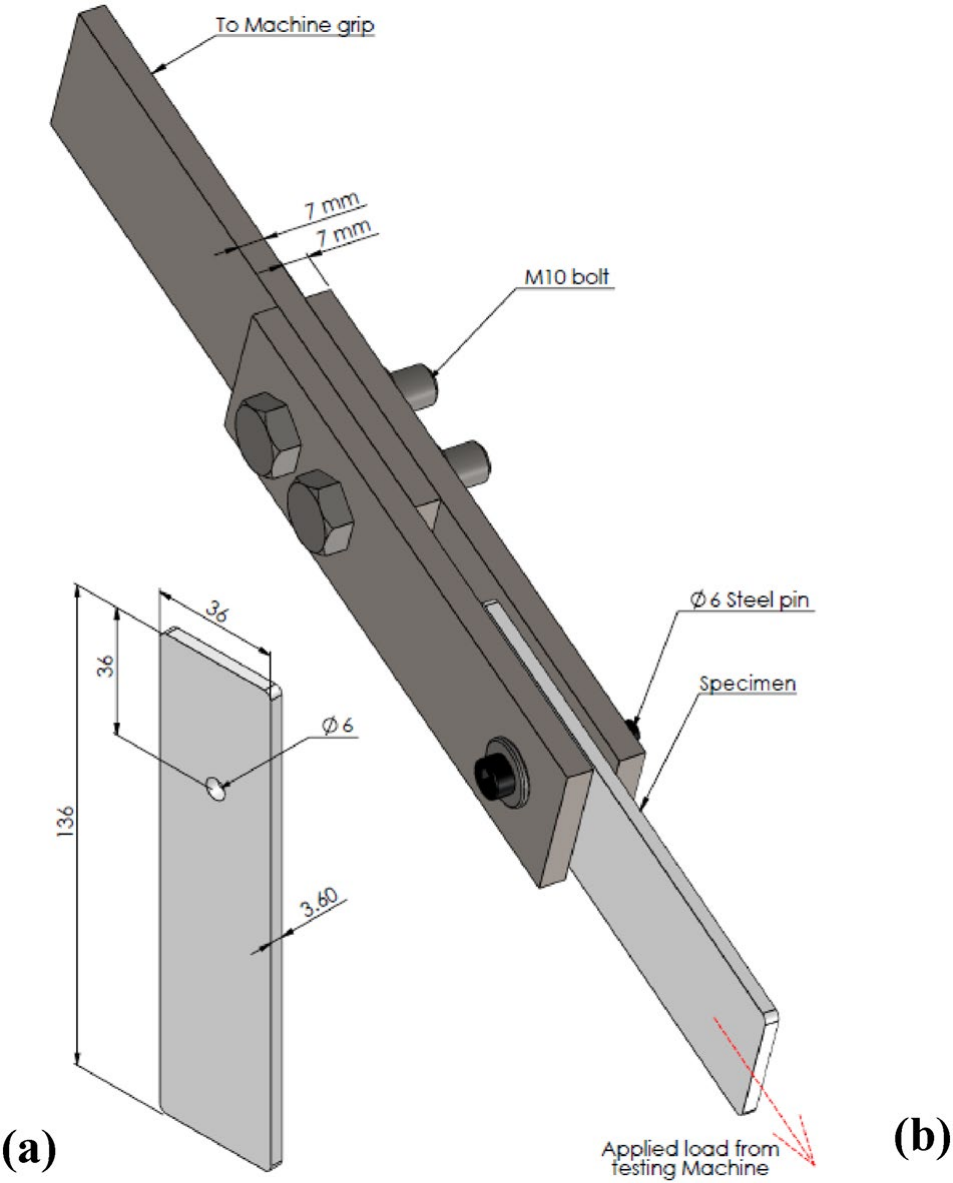
Bearing test specimen and fixture; (**a**) bearing specimen, (**b**) bearing fixture.

A series of pin-bearing ASTM D5961 tests were conducted on both molded-in and drilled specimens with different fiber contents using a universal testing machine (Testometric 200 kN) at room temperature. Standard test specimens were used to obtain bearing failure mode rather than net tension or shear-out modes that had lower loads associated with catastrophic fracture as recommended by previous studies^20,57–59^. The dimensions of the standard test specimen are illustrated in Fig. 2a where w/d = 6 and e/d = 6. The test fixture was manufactured from steel according to the geometry illustrated in Fig. 2b.

## Results and discussion

### Fiber length distribution (FLD)

Injection-molded fiber-reinforced PP composites of different fiber feedstock lengths (FFSL) of 12 and 24 mm were considered for the analysis. In injection molded parts, the number average fiber length (${L}_{n}$) and the weight average fiber length (${L}_{w}$) were obtained using the following relations;

4 $${L}_{n}=\frac{\sum {F}_{i}{L}_{i}}{\sum {F}_{i}} ,$$

and

5 $${L}_{w}=\frac{\sum {F}_{i}{{L}_{i}}^{2}}{\sum {F}_{i}{L}_{i}}.$$

The equations were proposed by Refs.^24,60^, where ${L}_{i}$ is the length of the ith fiber in the sample and ${F}_{i}$ is the frequency of fiber length ${L}_{i}$. The number average fiber length ${L}_{n}$ is always the smallest value and is strongly influenced by the presence of the amount of fibers and fragments. While the weight average fiber length ${L}_{w}$ is influenced by the presence of long fibers fraction. ${L}_{w}$ value is more expressive for the prediction of mechanical behavior^24,60^.

Several images of GF were obtained after complete burnout of the matrix in a muffle furnace at 570 °C for 4 h. The burnout test was conducted for all types of composites at different FFSL and wt%. The images were then analyzed using ImageJ software and + 500 measurements of GF were performed.

After analyzing the images it is clear that, the fiber lengths have dramatically decreased after the injection molding process^24,60–65^. This occurs due to the fibers go under massive shear stress through the injection process by the injection screw which leads to severe damages to the lengths of the fibers^63^.

**Figure 3:**
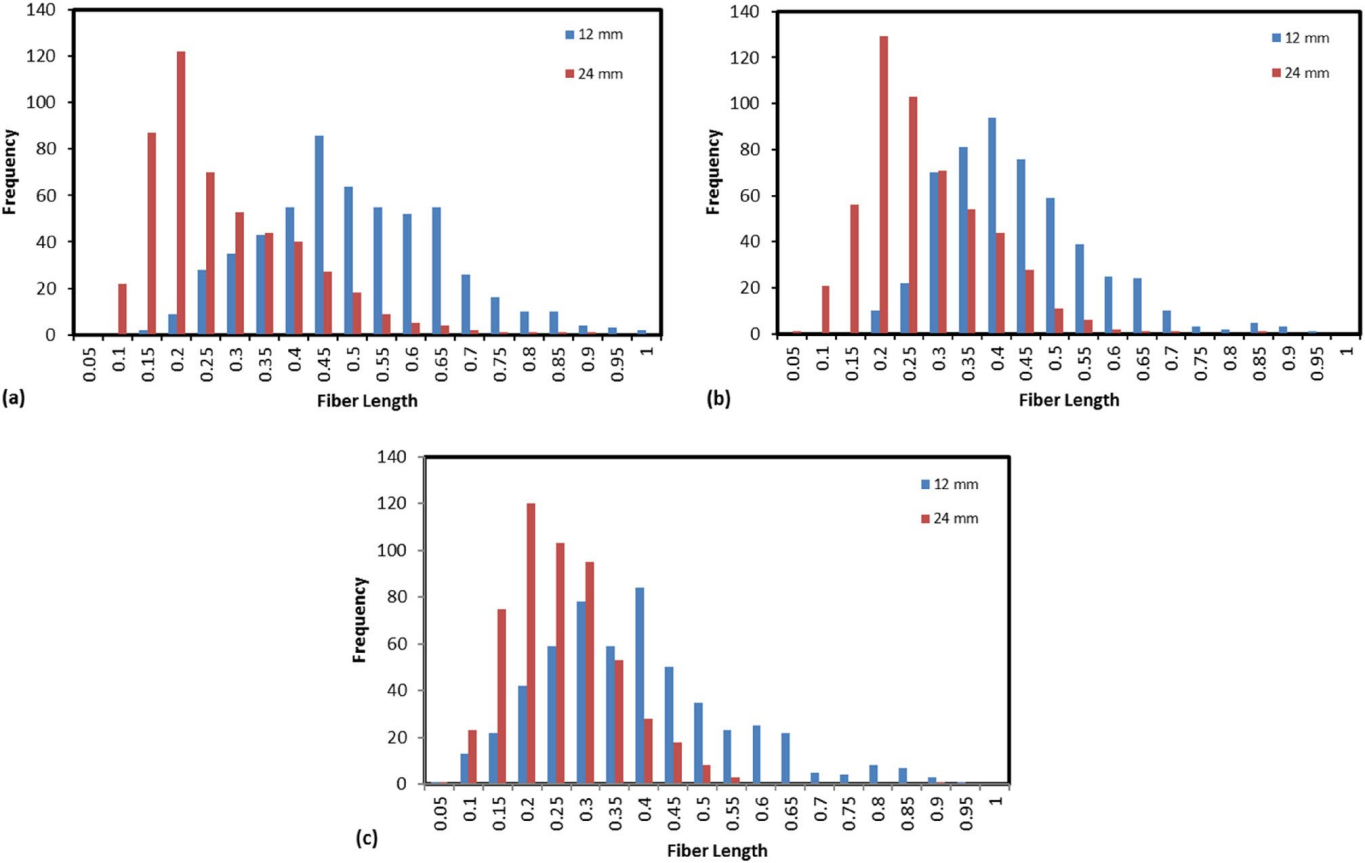
Fiber length distribution (FLD); (**a**) 10 wt%, (**b**) 20 wt%, and (**c**) 30 wt%.

Figure 3 shows Histograms describing the FLD of PP composites with different FFSL and wt%. The Histograms start from fiber lengths of 0.05 to 1 mm with a step of 0.05 mm based on the minimum and maximum values of the fiber lengths obtained from the measurements.

From the histograms shown in Fig. 3 it can be observed that, the composites with all compositions showed approximately normal distribution and higher frequencies were noticed to be shifted toward longer fibers in the case of specimens manufactured from 12 mm FFSL. The elevated frequencies of short fibers in 24 mm specimens will definitely reduce the values of both ${L}_{n}$ and ${L}_{w}$. The values of ${L}_{n}$ and ${L}_{w}$ for all specimens are tabulated in Table 6. Moreover, the frequency of fiber lengths tends to rapprochement between different FFSL by increasing fibers weight fractions from 10 to 30 wt%.

**Table 6:** The number average fiber length $${(L}_{n})$$ and weight average fiber length ($${L}_{w})$$ of GF-PP composites.

Weight fraction %	Feedstock fiber length (mm)	${L}_{n}$	${L}_{w}$	Aspect ratio
10	12	0.46	0.51	35.19
	24	0.28	0.34	21.16
20	12	0.44	0.48	33.61
	24	0.27	0.32	21.04
30	12	0.39	0.46	29.85
	24	0.26	0.29	19.85

Several studies^60–64^ discussed the influence of fibers weight fractions on the lengths of fibers in the injection molded glass fiber reinforced thermoplastics. These studies concluded that, the increase in fiber content leads to a decrease in the fiber lengths in the resulting composite. Kumar et al.^60^ related this reduction in fiber lengths to the increased damage occurred to the fibers due to elevated interaction between fibers at higher concentrations in the composite. They also showed that, both ${L}_{n}$ and ${L}_{w}$ increase as FFSL increases for FFSL up to 9 mm, further increase in FFSL more than 9 mm has a reverse effect where both ${L}_{n}$ and ${L}_{w}$ decrease.

**Figure 4:**
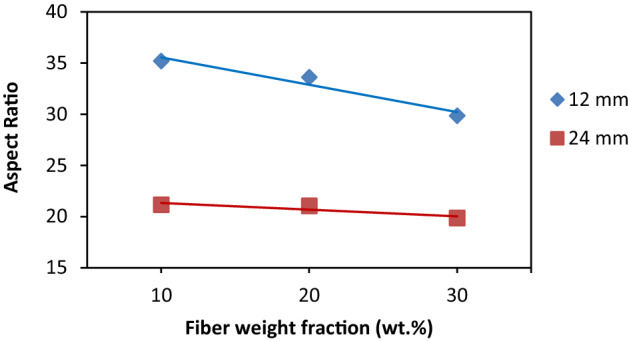
Relation between fiber weight fraction and fiber aspect ratio.

It has been observed from Fig. 4 that, the average fiber length and the aspect ratios after injection molding decrease as the FFSL increased from 12 to 24 mm. For example, ${L}_{w}$ of 10 wt% was decreased by 150% using 24 mm GF when compared with 12 mm GF. Therefore, the increase of FFSL more than 12 mm may lead to a significant decrease in the fiber aspect ratio as shown in Fig. 4. It is also observed from Table 6 that, the increase in fiber weight fraction leads to a slight decrease in the average fiber length as previously observed by Refs.^60–64^. Throughout this work, based on the above results, FFSL of 12 mm and 24 mm will be referred to as “long fiber/Polypropylene (LFPP)” and “short fiber/Polypropylene (SFPP)”, respectively.

### BS of molded-in holes

**Figure 5:**
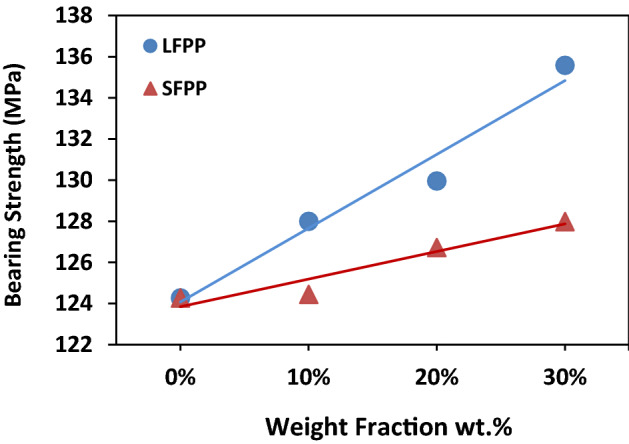
BSs of molded-in holes specimens with different weight fractions.

Figure 5 represents the relationship between BS of the GFR/PP composites with different wt% and FFSL. The figure shows a decrease in the BS of SFPP composites than LFPP composites. The observed decrease in BS starts from 2.85% at 10 wt% up to 5.95% at 30 wt%. The decrease in BS may be occurred due to the decreased aspect ratios of the fibers in the obtained composites at increased FFSL as illustrated in Fig. 4.

It can also be noted from Fig. 5 that, the fiber content and length in the matrix have a strong effect on the BS of GFR/PP composites. For both SFPP and LFPP the BS increases as the fiber wt% increases with an improvement of 9% for L3012 specimen above L00. While for SFPP, BS increases by only 3% from the L00 specimen to L3024 one. The increase of BS due to the increase of fiber wt% is expected as the strength of the GF reinforcement material is significantly greater than PP, increasing the wt% of GF directly improves the BS of composites as shown in Fig. 5. A similar result was reported by Subramanian and Senthilvelan^25^ where the BS of leaf spring made from GFR/PP was higher than that made of un-reinforced PP. Also, as the fiber length increased BS increased. Moreover, Asi^66^ showed that BS of GFRE firstly increased as linear densities of woven fabric increased (which is an indication of the increase in fiber content) and then decreased with an extra increase in woven fabric linear densities as a result of elevated void content and crimp levels of the obtained composite.

The variety in the percentage of improvement for the BS from L00 to L3012 and L3024 which is three times higher in L3012 (LFPP) than L3024 (SFPP) may be related to the difference in average fiber length (aspect ratio) between them, as found in previous studies^24,60^. Where Subramanian et al.^24^ and Kumar et al.^60^ found that the strength of composite increases as the mean fiber length increases. Kumar et al.^60^ noticed that the strength of composite depends mostly on the fiber aspect ratio (or fiber length) more than fiber content, and the reduced strength of the composite caused by decreased mean fiber length almost offsets the increased composite strength caused by higher fiber content.

**Figure 6:**
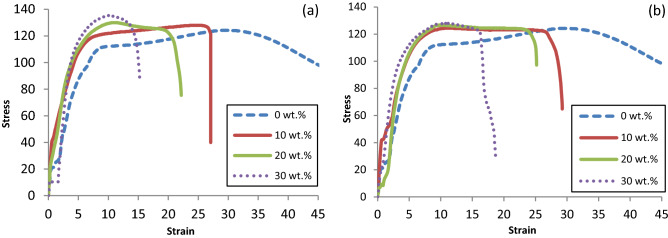
Stress–strain curves of; (**a**) LFPP and neat PP, (**b**) SFPP and neat PP.

Figure 6a,b show the stress–strain curves for specimens with different GF wt% for both LFPP and SFPP, respectively. As already discussed in Fig. 5, stress strain curves in Fig. 6 also show the enhancement of the bearing strengths as higher weight fractions of fibers introduced to the matrix and further enhancements of BS for specimens with longer fibers. It is also observed from Fig. 6 that, failure strains of the GF/PP composites are inversely proportional to fiber wt% due to the reduced elongation of GF compared to PP’s elongation as mentioned in Tables 1 and 2.

### BS of drilled holes

The experimental results of measured BS, the corresponding values of S/N ratios, and experiment code for each trial are shown in Table 7, for both LFPP and SFPP sorted respectively from experiment 1 to experiment 16.

**Table 7:** Design and experimental results of the L16 orthogonal array.

Experiment code	LFRPP		SFRPP	
	BS	S/N ratios	BS	S/N ratios
W1S1F1	126.46	42.04	126.46	42.04
W1S2F2	122.66	41.77	122.66	41.77
W1S3F3	119.67	41.56	119.67	41.56
W1S4F4	124.24	41.89	124.24	41.89
W2S1F2	128.18	42.16	127.92	42.14
W2S2F1	124.96	41.94	120.62	41.63
W2S3F4	124.74	41.92	121.17	41.67
W2S4F3	127.13	42.08	125.66	41.98
W3S1F3	134.28	42.56	129.22	42.23
W3S2F4	129.38	42.24	125.61	41.98
W3S3F1	125.61	41.98	121.06	41.66
W3S4F2	128.49	42.18	121.78	41.71
W4S1F4	134.07	42.55	131.00	42.35
W4S2F3	132.06	42.42	121.52	41.69
W4S3F2	134.45	42.57	122.87	41.79
W4S4F1	136.86	42.73	123.23	41.81

Tables 8 and 9 represent the rank of the effect of each factor on response parameter (BS) for LFPP and SFPP, respectively, by Taguchi analysis using Minitab 17 software. Minitab software assigns ranks based on Delta values; rank 1 referred to the highest value of Delta, rank 2 represents the second-highest value of Delta, and so on, to indicate the corresponding effect of each factor on the response (BS). For LFPP, the weight fraction is the most effective factor on the BS, followed by speed, and then the feed. For SFPP, the most effective factor on BS is the speed, followed by feed, and then weight fraction. Different ranks for the factors between LFPP and SFPP are noticed.

**Table 8:** Response table for S/N ratios and BS of drilled LFPP.

Level	Weight fraction		Speed		Feed	
	S/N ratio	BS	S/N ratio	BS	S/N ratio	BS
1	41.81	123.3	42.33	130.7	42.17	128.5
2	42.02	126.3	42.09	127.3	42.17	128.4
3	42.24	129.4	42.01	126.1	42.16	128.3
4	42.56	134.4	42.22	129.2	42.15	128.1
Delta	0.75	11.1	0.32	4.6	0.02	0.4
Rank	1		2		3

**Table 9:** Response table for S/N ratios and BS of drilled SFPP.

Level	Weight fraction		Speed		Feed	
	S/N ratio	BS	S/N ratio	BS	S/N ratio	BS
1	41.81	123.3	42.19	128.6	41.79	122.8
2	41.85	123.8	41.77	122.6	41.85	123.8
3	41.89	124.4	41.67	121.2	41.87	124
4	41.91	124.7	41.85	123.7	41.97	125.5
Delta	0.1	1.4	0.52	7.4	0.18	2.7
Rank	3		1		2	

**Figure 7:**
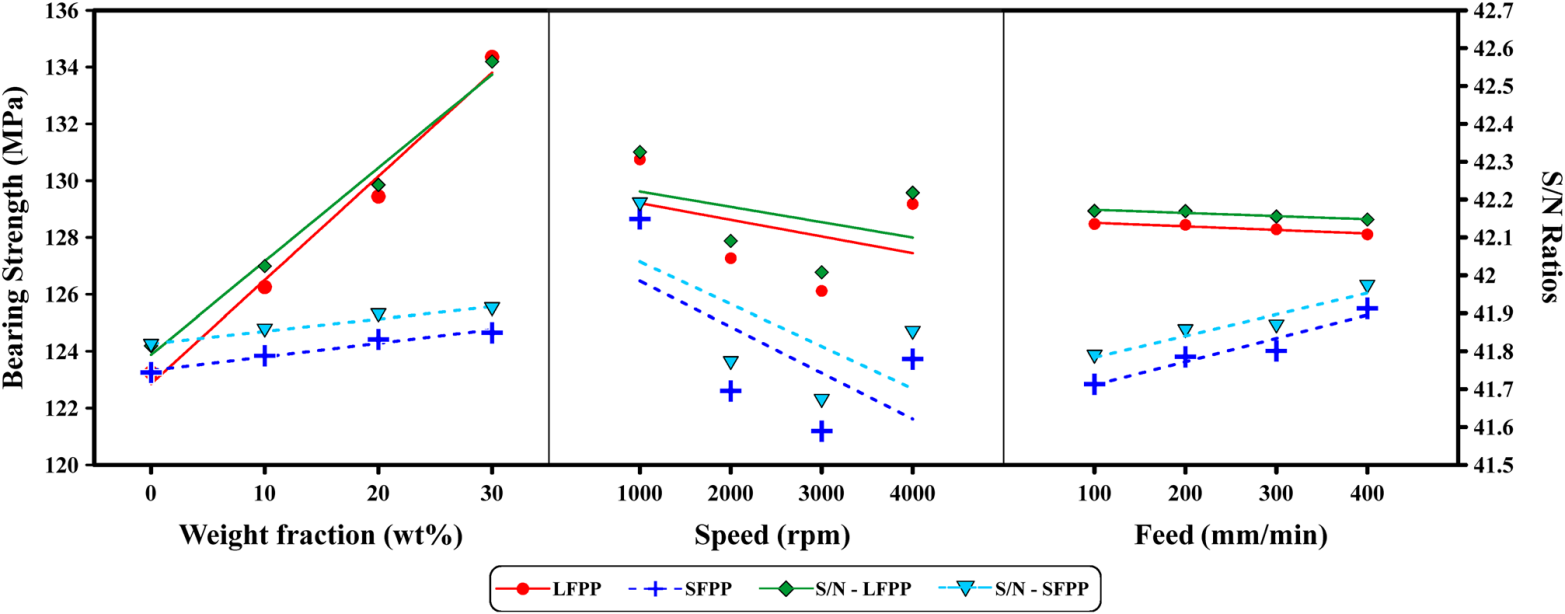
Mean effect plot for LFPP and SFPP for BS and S/N values.

ANOVA general linear model along with One-way ANOVA was obtained to describe the response of each factor where equal variances for the analysis were assumed. Results of the ANOVA general linear model and one-way ANOVA are summarized in Tables 10 and 11, respectively. Comparing the p-value for each factor with significance level (α = 0.05) indicates that, for LFPP weight fraction has p-value less than significance level α (p-value = 0.003), Table 10. While feed and speed have p-values higher than α. However, in the case of SFPP, speed has a p-value below the significance level α (p-value = 0.005). While feed and weight fractions have p-values higher than α. The weight fraction in the case of LFPP is the most significant factor affecting the BS. BS increases as the weight fraction increases with a maximum improvement in BS of 9% for 30 wt% over neat PP. While speed and feed have an insignificant effect as the slope gradient is very small as shown in Fig. 7. Different results are obtained for SFPP, where speed is the significant factor affecting BS. BS decreases as the speed increases from 1000 to 4000 rpm. On the other hand, weight fraction and feed have insignificant effects on BS with a very small slope, as shown in Fig. 7. The results of ANOVA agree well with the results obtained using Minitab 17 software. The absence of weight fraction influential effect on BS in the case of SFPP may be due to the reduced weighted average fiber length which clears the field to the machining parameters to show their effect on the BS of GFR/PP composites represented by spindle speed.

**Table 10:** ANOVA general linear model for drilled PP and GFR/PP under different drilling conditions.

Source	DF	LFPP				SFPP			
		Adj SS	Adj MS	F-value	p-value	Adj SS	Adj MS	F-value	p-value
Wt%	3	270.50	90.17	16.49	**0.003**	4.702	1.57	0.49	**0.703**
Speed	3	50.4	16.8	3.07	**0.112**	126.09	42.03	13.1	**0.005**
Feed	3	0.339	0.113	0.02	**0.996**	14.54	4.85	1.51	**0.305**
Error	6	32.806	5.4676			19.252	3.209		
Total	15	354.04				164.578			

Significant values are in bold.

**Table 11:** One-way ANOVA for drilled PP and GFR/PP under different drilling conditions.

Source	DF	LFPP				SFPP			
		Adj SS	Adj MS	F-value	p-value	Adj SS	Adj MS	F-value	p-value
Wt%	3	270.50	90.166	12.95	**0.000**	4.702	1.567	0.12	**0.948**
Error	12	83.54	6.962			159.876	13.323		
Total	15	354.04				164.578			
Speed	3	50.40	16.80	0.66	**0.590**	126.09	42.029	13.10	**0.000**
Error	12	303.64	25.30			38.49	3.208		
Total	15	354.04				164.58			
Feed	3	0.339	0.1131	0	**1.000**	14.54	4.845	0.39	**0.764**
Error	12	353.70	29.475			150.04	12.503		
Total	15	354.04				164.58			

Significant values are in bold.

### BS of molded-in holes compared to machined holes

#### BS of molded-in vs drilled holes

**Figure 8:**
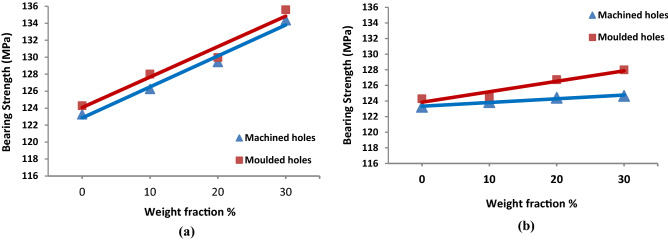
BS of molded-in holes vs. machined holes; (**a**) LFPP, (**b**) SFPP.

Figure 8a,b show the difference between BS of specimens with molded-in holes and machined holes for both LFPP and SFPP specimens, respectively. It is clear from the figure that, BS of the molded-in holes is slightly better than those of machined holes for all weight fractions and fiber lengths. Collateral damages to the drilling process are playing a major role in the reduced BS of specimens with drilled holes. The slope at Fig. 8a shows similar behavior for molded-in and machined specimens for LFPP, where they have the same rate of increase of BS along with the increase of fiber weight fraction. The average increase in BS for specimens with molded-in holes is about 1% above drilled holes specimens. Whereas the slope at Fig. 8b shows a slightly higher rate of increase of BS along with an increase in fiber weight fraction for molded-in hole specimens compared to the machined hole specimens for SFPP. The increase in BS for specimens with molded-in holes ranges from 0.8% for neat PP to 2.6% for 30 wt% GF over drilled holes specimens.

The bearing test results of Hufenbach et al.^30^ showed that textile-reinforced thermoplastics molded-in holes were able to withstand greater loads compared to the drilled configuration.

#### Analysis of specimens’ failure due to bearing test

**Figure 9:**
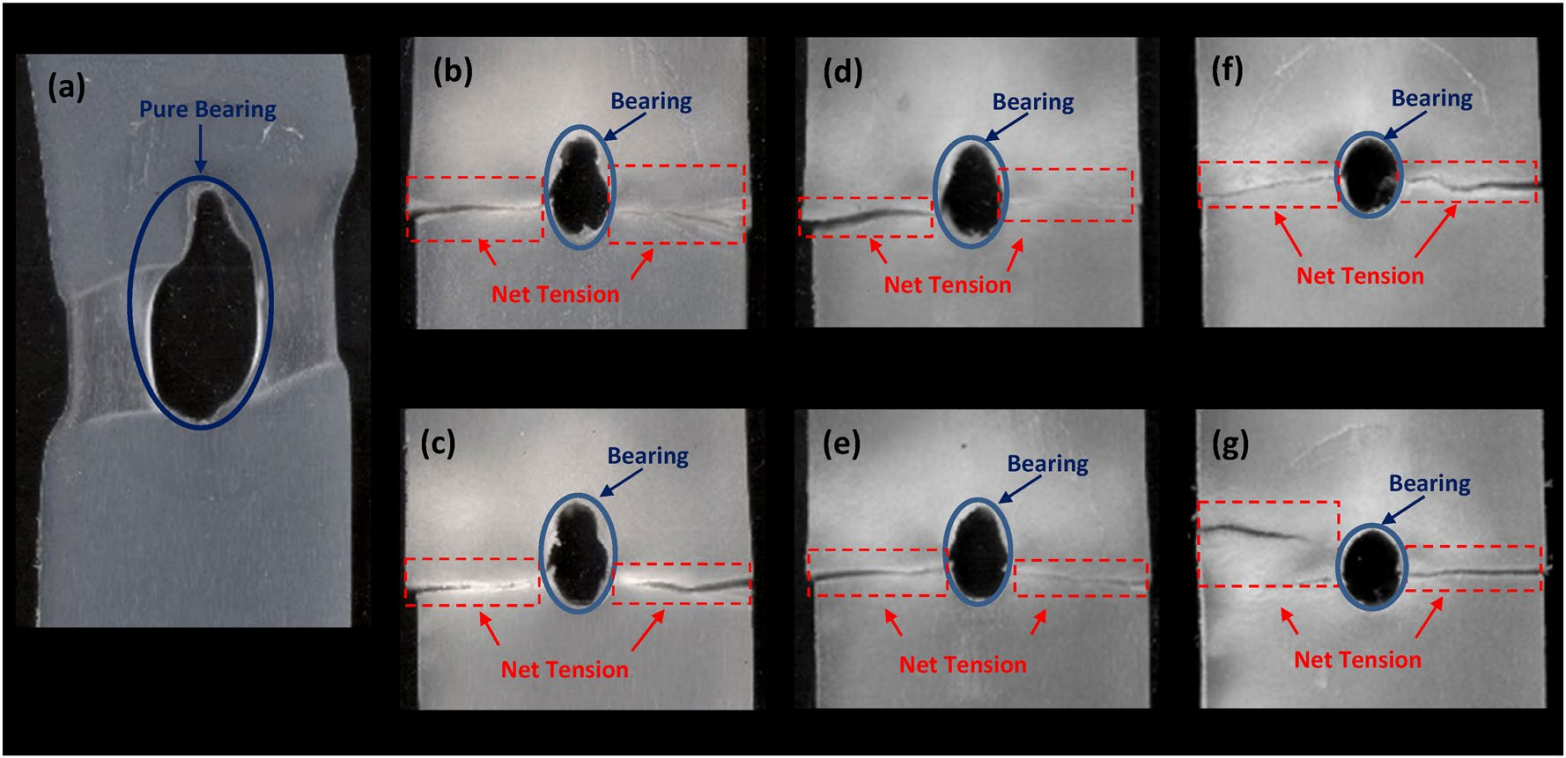
Failure morphologies of molded-in hole specimens tested in bearing; (**a**) Neat PP, (**b**) 10 wt% GF (12 mm initial length) + 90 wt% PP, (**c**) 10 wt% GF (24 mm initial length) + 90 wt% PP, (**d**) 20 wt% GF (12 mm initial length) + 80 wt% PP, (**e**) 20 wt% GF (24 mm initial length) + 80 wt% PP, (**f**) 30 wt% GF (12 mm initial length) + 70 wt% PP, and (**g**) 30 wt% GF (12 mm initial length) + 70 wt% PP.

Experimentally, the mechanically fastened joints fail under four basic mechanisms; net tension, shear out, cleavage failure, and bearing failure. The net tension, shear out, and cleavage failure modes are not desirable due to the catastrophic nature of final failure^20^. Bearing failure, which is characterized by a progressive decrease in applied load, is considered the ideal failure mode^38,67^. the failure damage of fiber-reinforced material might be attributed to the matrix cracking, fiber fracture, fiber-matrix interfacial debonding, and their combinations^68^. In this section, the failure mode of bolted joint composites was evaluated by observing the failure surface. Figure 9 shows the failure morphologies of molded-in specimens with different weight fractions and fiber lengths tested in bearing. From Fig. 9 it is noticed that, two failure modes have occurred in the present work as a result of the bearing test. The first failure mode is the pure bearing mode which is represented in neat PP specimen (L00) as shown in Fig. 9a. The second failure mode is the mixed failure mode (net-tension/bearing mode) of GFR/PP composite specimens as shown in Fig. 9b–g. Similar failure modes between different fiber lengths are noticed, while the bearing capacity decreases as the fiber weight fraction increases. Therefore, for L3012 and L3024 specimens shown in Fig. 9f,g bearing failure has barely occurred, while impressive bearing capacity is obtained by neat PP as indicated in Fig. 9a.

**Figure 10:**
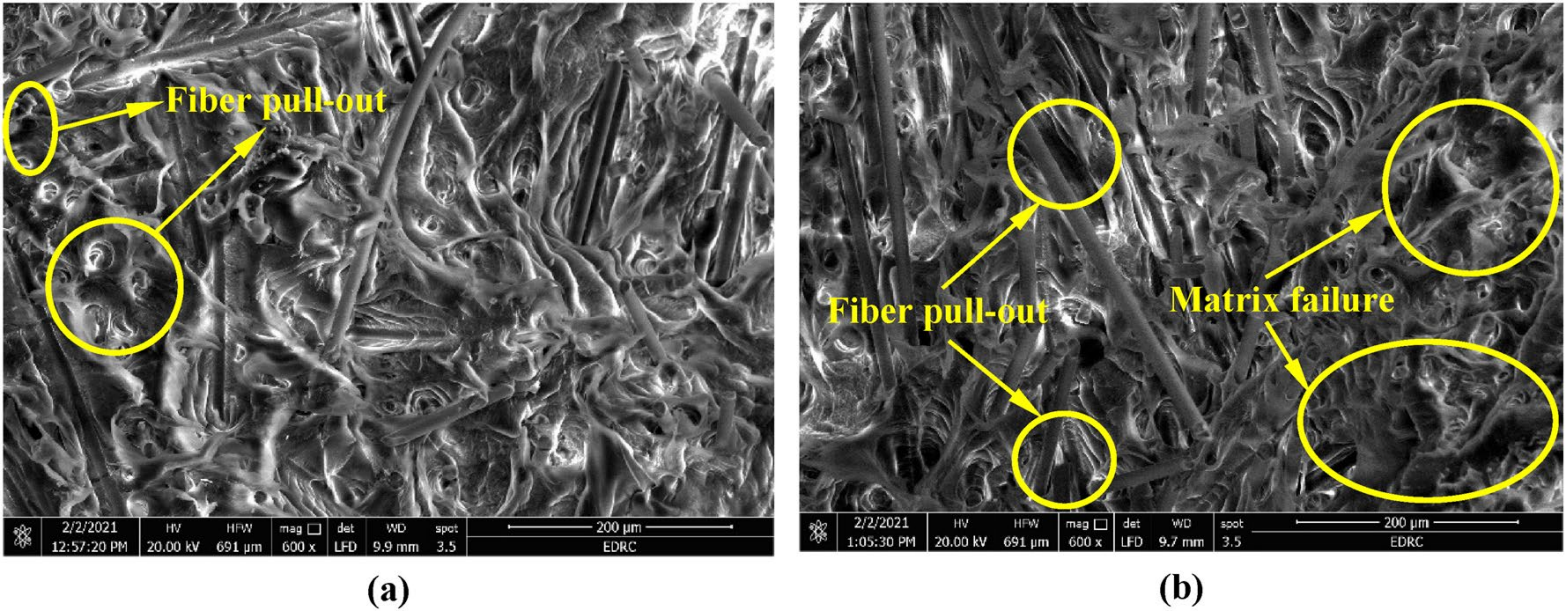
SEM images of a cross-section of the fractured zone of specimens; (**a**) 10 wt% GF (12 mm initial length) + 90 wt% PP and (**b**) 30 wt% GF (12 mm initial length) + 70 wt% PP.

The SEM micrographs of the fracture zone of L1012 and L3012 specimens are shown in Fig. 10a,b, respectively. The brittle fracture of the matrix is more obvious in the L3012 specimen than the L1012 specimen, which is proportionate with the brittle nature of the stress–strain curves for the L3012 specimen as shown before in Fig. 6. Moreover, it can be noticed from Fig. 10 that apparently a large number of fibers are pulled out from the matrix in both specimens due to specimen fracture.

**Figure 11:**
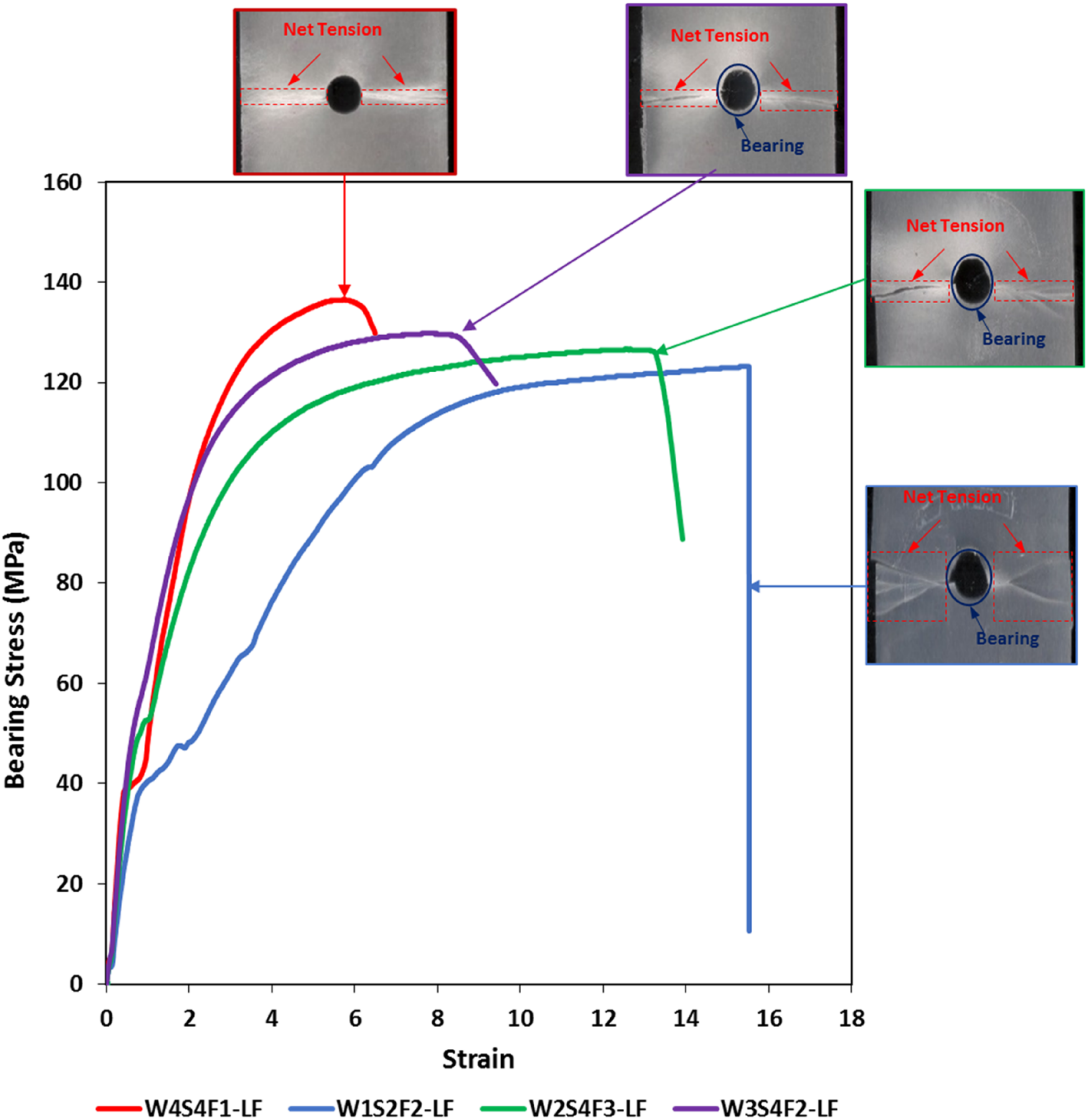
Failure morphologies and bearing stress–strain curves of drilled hole specimens.

Figure 11 includes the stress–strain relationship besides the failure morphology of some machined holes specimens. A mixed failure mode (net-tension/bearing mode) has occurred for all specimens having machined holes except for 30 wt% GF specimens which failed under net-tension mode only as shown in Fig. 11. The change of failure mode between molded-in and drilled specimens may be attributed to the damages associated with the drilling process.

## Conclusions

An experimental and statistical analysis for BS associated with GFR/PP composites manufactured by injection molding technique with either molded-in or drilled holes at various drilling conditions are presented in this study. The obtained results may be summarized as follow;

- The BS of specimens injected with longer FFSL was found to have a lower BS than shorter ones due to the observed decrease in weight average fiber length in the produced specimen after the injection molding process. The observed decrease in BS starts from 2.85% at 10 wt% up to 5.95% at 30 wt% GFR/PP.
- For specimens with molded-in holes, for both LFPP and SFPP, BS increases as the fiber weight fraction increases. For LFPP, an improvement of 9% for L3012 specimens above L00 is obtained. While for SFPP, BS increases by only 3% from L00 specimen to L3024 one.
- For specimens with drilled holes, results obtained from ANOVA and Taguchi analysis indicated that the effects of the machining conditions and weight fraction on BS were different between LFPP and SFPP specimens; for LFPP, the most significant factor was the weight fraction while the drilling conditions (speed and feed) were found to be less significant. However, for SFPP spindle speed was found to be the most significant factor, followed by feed, while the weight fraction has the least effect.
- The increase in weight fraction leads to an increase in BS for both molded-in and machined hole specimens.
- BS of the molded-in holes is slightly better than those of machined holes for all used weight fractions and fiber lengths; for LFPP, the average increase in BS for specimens with molded-in holes is about 1% above drilled holes specimens. Whereas for SFPP, the increase in BS for specimens with molded-in holes ranges from 0.8% for neat PP to 2.6% for 30 wt% GFR/PP over drilled holes specimens.
- Morphology analysis for fractured specimens indicated that; for molded-in hole specimens, plain PP specimens failed under pure bearing failure mode. While GFR/PP specimens failed under bearing and net tension mixed-mode failure. For machined hole specimens, all specimens failed under bearing and net tension mixed-mode failure, except for 30 wt% GFR/PP specimens that failed under net tension failure mode only.

## Data Availability

The authors declare that all data generated or analyzed during this study are included in this published article.
